# Supplementary figures and images for: Gluconobacter dominates the gut microbiome of the Asian palm civet Paradoxurus hermaphroditus that produces kopi luwak
Source: PeerJ. 2020 Jul 30;8:e9579. doi: 10.7717/peerj.9579 (PMC7396140; doi:10.7717/peerj.9579)

# maximum growth temperature

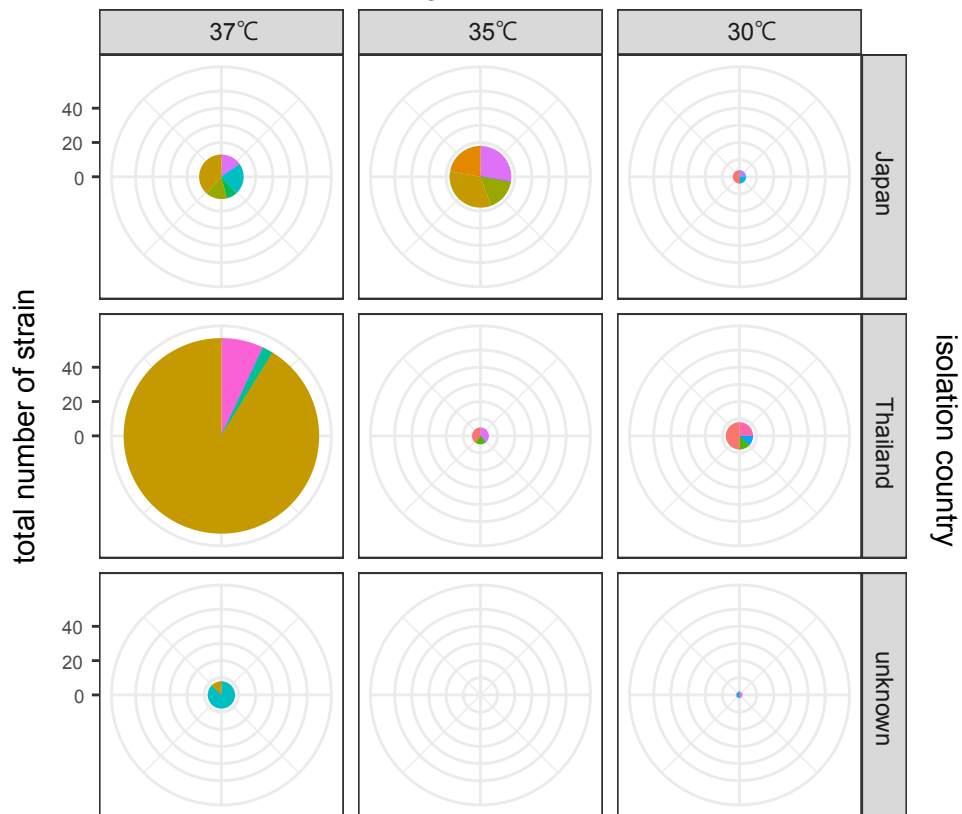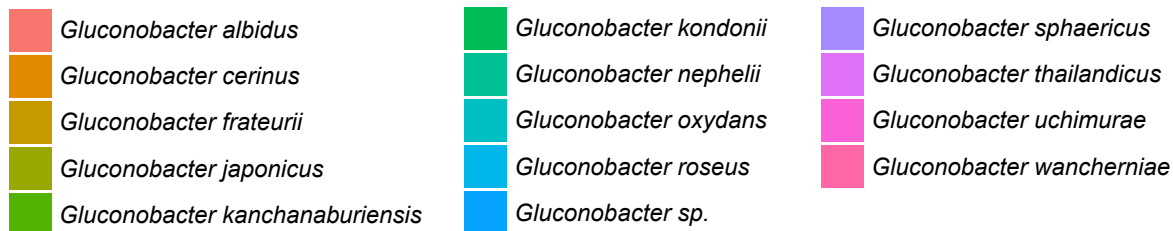

Supplement: Supplemental Information 2 [file peerj-08-9579-s002.pdf]
