# Supplementary material for: Gluconobacter dominates the gut microbiome of the Asian palm civet Paradoxurus hermaphroditus that produces kopi luwak
Source: PeerJ. 2020 Jul 30;8:e9579. doi: 10.7717/peerj.9579 (PMC7396140; doi:10.7717/peerj.9579)
Supplement: Supplemental Information 1 [file peerj-08-9579-s001.pdf]

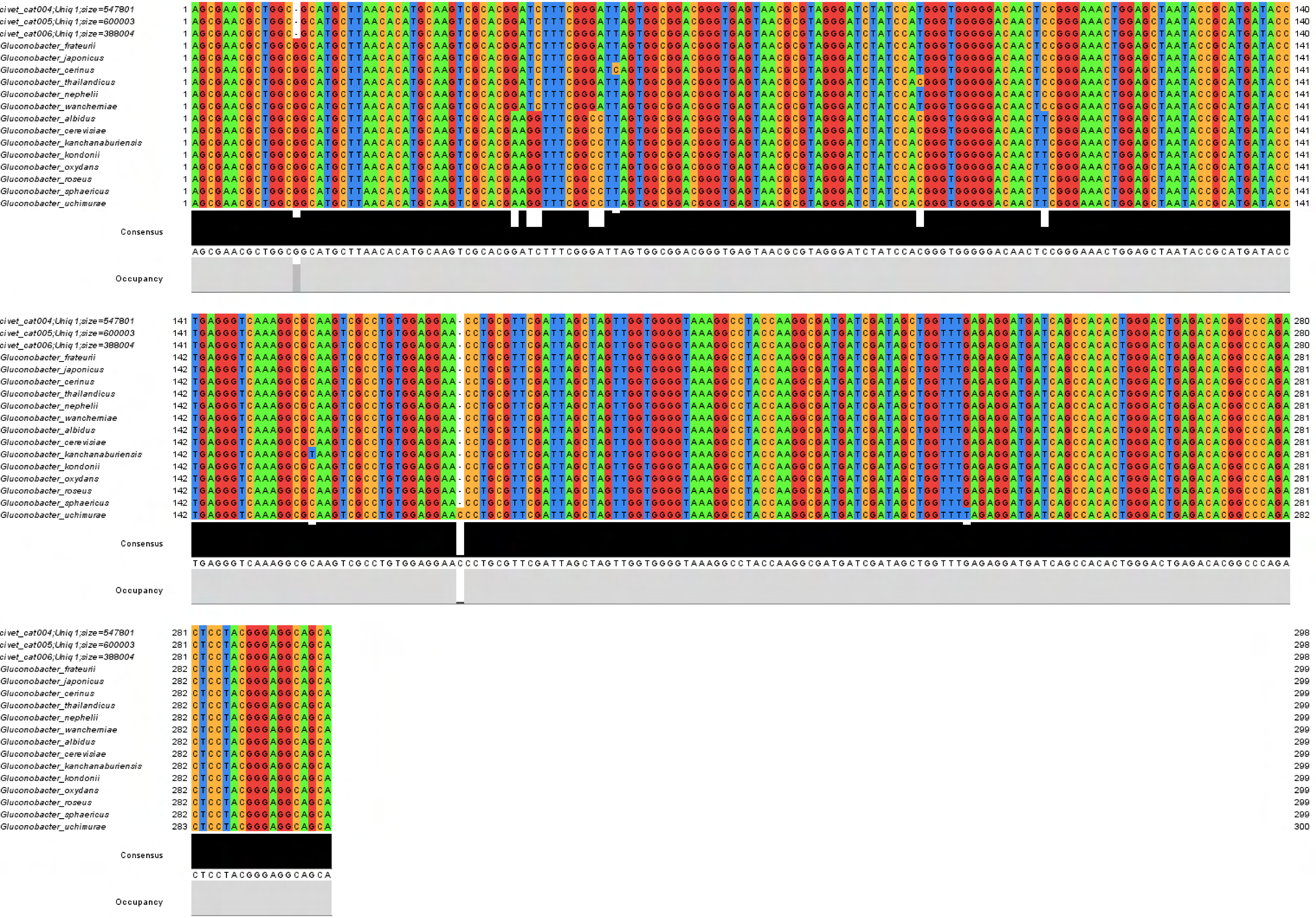
civet_cat004;Uniq1;size=547801
civet_cat005;Uniq1;size=600003
civet_cat006;Uniq1;size=388004
Gluconobacter_frateurii
Gluconobacter_japonicus
Gluconobacter_cerinus
Gluconobacter_thailandicus
Gluconobacter_nephelii
Gluconobacter_wancherniae
Gluconobacter_albidus
Gluconobacter_cerevisiae
Gluconobacter_kanchanaburiensis
Gluconobacter_kondonii
Gluconobacter_oxydans
Gluconobacter_roseus
Gluconobacter_sphaericus
Gluconobacter_uchimurae
Consensus
AGCGAACGCTGGCGGCATGCTTAACACATGCAAGTCGCACGGATCTTTCGGGATTAGTGGCGGACGGGTGAGTAACGCGTAGGGATCTATCCACGGGTGGGGGACAACTCCGGGAAACTGGAGCTAATACCGCATGATACC
Occupancy
Consensus
TGAGGGTCAAAGGCGCAAGTCGCCTGTGGAGGAACCCTGCGTTCGATTAGCTAGTTGGTGGGGTAAAGGCCTACCAAGGCGATGATCGATAGCTGGTTTGAGAGGATGATCAGCCACACTGGGACTGAGACACGGCCCAGA
Occupancy
Consensus
CTCCTACGGGAGGCAGCA
Occupancy
